# Association of Insomnia in Patients with Chronic Kidney Disease on Maintenance Hemodialysis

**DOI:** 10.7759/cureus.9520

**Published:** 2020-08-02

**Authors:** Omer Farooq Rehman, Uzma Rauf, Maryam Rauf, Sana Aziz, Ahmad Faraz, Farah Anum Jameel

**Affiliations:** 1 Urology, Armed Forces Institute of Urology, Rawalpindi, PAK; 2 Nephrology, Khyber Teaching Hospital, Peshawar, PAK; 3 Plastic Surgery, Burns and Plastic Surgery Unit, Hayatabad Medical Complex Medical Teaching Institute (HMC-MTI), Peshawar, PAK; 4 Internal Medicine, Hayatabad Medical Complex (HMC), Peshawar, PAK; 5 Trauma and Orthopaedics, Leeds Teaching Hospitals NHS Trust, Leeds, GBR; 6 Nephrology, Jinnah Postgraduate Medical Centre, Karachi, PAK

**Keywords:** chronic kidney disease, anxiety, insomnia, hemodialysis

## Abstract

Objective

To determine the frequency of insomnia in patients with chronic kidney disease (CKD) on maintenance hemodialysis (HD)

Study design

Cross-sectional (descriptive)

Study duration

From July 20, 2019, to January 20, 2020

Study settings

Department of Nephrology, Khyber Teaching Hospital, Peshawar

Materials and methods

A total of 148 patients diagnosed with CKD and on maintenance HD were selected for the study in a consecutive sampling manner and checked for insomnia.

Results

Out of 148 patients included in the study, there were 64.9% male and 35.1% female patients. The mean duration of CKD (months) was 13.9 ± 6.3. The mean number of sessions for hemodialysis done in all patients was 16.8 ± 5.3. On careful interviewing of the patient, difficulty in falling asleep was recorded in 28.4%, difficulty in staying asleep in 41.9%, problems in waking up early in 34.5%, and insomnia interfering in routine life activities in 28.4%. Overall, insomnia was recorded in 36.5% of patients and was found to have a significant correlation with the number of dialysis sessions (p-value 0.000).

Conclusion

Insomnia is a frequent disorder associated with CKD patients on maintenance HD. There are very few studies establishing its pathogenesis and risk factors. We recommend further multicenter studies to detect the course of insomnia in association with CKD on HD and its potential impact on the overall quality of life of patients with CKD.

## Introduction

Patients with kidney disorders are blighted with various types of sleep problems. Many a time, healthcare professionals fail to recognize the kind of sleep disorder unless they know the common sleep-related issues in accordance with chronic kidney disease (CKD) [1]. Various sleep disorders are associated with CKD, which include insomnia, sleep-related breathing disorders, hypoventilation, central sleep apnea, central disorders of hypersomnolence, circadian rhythm sleep-wake disorders, parasomnias, and sleep-related movement disorders [2].

American Academy of Sleep Medicine (AASM) defined insomnia as a repeated difficulty with sleep initiation, duration, consolidation, or quality that happens despite adequate opportunity and circumstances for sleep and results in some form of daytime impairment [3]. Patients with insomnia have been observed to have problems with falling asleep, continuing sleep cycle, and/or unwanted sustained periods of wakefulness during sleep [4]. Studies have shown cognitive and physiological risk factors for Insomnia. Maung et al. found that metabolic disturbances secondary to chronic kidney disease may influence abnormalities in sleep behavior [5]. Patients with renal disorders, CKD, or end-stage renal disease (ESRD) are associated with progression to various sleep abnormalities.

Various studies show the impact of kidney dysfunction on sleep. Hanly et al. observed that there is a direct linear correlation between CKD or ESRD and insomnia. Specifically, a worsening CKD is directly associated with worsened insomnia [6]. Furthermore, a descriptive study published by Aggarwal et al. [7] observed insomnia among 60% of patients who underwent hemodialysis (HD), whereas other studies report the frequency of insomnia as 86.5% [8] and 45% [9].

To further describe the specific etiology, Rayner proposed a direct relationship between decreased orexin levels to a decline in wakefulness (narcolepsy) [10]. He further proposed that in the case of CKD or end-stage renal disease (ESRD) levels of orexin are significantly high, causing lost sleep [11]. Also, psychiatric overtones (depression, psychosis, and insomnia) can ensue due to hypercalcemia associated with CKD or ESRD [12].

The previously published study shows a variable proportion of insomnia in CKD but also gives a mixed type of sample size used [5]. Therefore, the current study has been set up to investigate the magnitude of insomnia among patients with co-morbid of CKD or ESRD on hemodialysis in our ethnic groups. We will request further studies over the usefulness of interventions designed to alleviate sleep disorders in these patients.

## Materials and methods

This cross-sectional (descriptive) study was conducted at the nephrology department of Khyber Teaching Hospital, Peshawar. Following approval from the hospital ethical and research committee, this study was conducted over a period of six months, from July 2019 till January 2020.

The sample size was 148, 42.9%, i.e., 15 proportion of insomnia in CKD patients on hemodialysis with a 95% confidence level and an 8% margin of error using the World Health Organization (WHO) sample size calculator. The sampling technique was non-probability consecutive sampling.

Inclusion criteria

CKD patients who underwent a minimum of 10 hemodialysis sessions

Exclusion criteria

1. Patients previously diagnosed with insomnia, anxiety, or depression

2. Patients with a history of emotional trauma, sudden loss of assets, or in a near relative, in the previous six months

All patients meeting the selection criteria were included in the study through the outpatient department (OPD) or the dialysis center. Written informed consent was taken from all the patients and they were subjected to a detailed history and clinical examination and underwent an in-depth interview in an undisturbed environment to detect findings suggestive of insomnia. Findings were recorded in the presence of a consultant nephrologist and an attending psychiatrist, with both having a minimum of five years of consultancy experience.

After recording the necessary demographics, care was taken during the extraction of information from all patients to avoid responder bias. Confounders and other biases were controlled by strictly following the exclusion criteria.

Data were recorded in Statistical Package for the Social Sciences (SPSS) version 20 (IBM Corp, Armonk, NY). Descriptive statistics were used to analyze data, where mean and standard deviation was calculated for quantitative variables like age, duration of CKD, and the number of hemodialysis sessions (HDs) done in the past. Whereas, frequency and percentages were calculated for categorical variables (gender and insomnia). Insomnia was stratified according to the age, gender, duration of CKD, and episodes of hemodialysis, to establish the effect modifiers using the chi-square test with a P-value of < 0.05 as significant and the independent-sample t-test used for comparing means between the groups.

## Results

A total of 148 subjects were included in the present study, which had CKD as per the operational definition. Their demographic and clinical data have been depicted in Table 1. The age of the included patients ranges between 18 and 60 years. Male outnumbered females (64.9% vs 35.1%) in our study. Age did not impact insomnia with a p-value of 0.362, whereas gender did not show any significant correlation (p-value 0.713) as well, as given in Table 1. After applying chi-square, we found no significant association between insomnia and the duration of CKD (p-value 0.940).

**Table 1:** Demographic characteristic of patients

No. of patients	148	p-value	
Gender			
Male	96 (64.9%)	0.713	
Female	52 (35.1%)		
Age range			
18 to 30 years	26	0.713	
> 30 to 40 years	47		
>40 to 50 years	31		
> 50 to 60 years	44		
Newly diagnosed patients of Insomnia on hemodialysis			
54 (36.5%)			
Hemodialysis sessions			
11-15	81		
>15 - 20	12		
>20 - 25	55		
DURATION OF CKD	With insomnia	Without insomnia	0.940
3 to 9 months	15(38.5%)	24 (61.5%)	
> 9 to 16 months	18 (36.7%	31 (63.3%)	
> 16 to 24 months	21(35.0%)	39 (65%)	

Insomnia fulfilling the International Classification of Sleep Disorders-2 (ICSD-2) criteria [12] was found among 54 patients (36.5%). On further questioning the subjects, we found that difficulty in initiation of sleep was recorded among 28.4%, problems in maintaining sleep state in 41.9%, problems in waking up early in 34.5%, and sleep deprivation interfering in routine life activities in 28.4%. We stratified insomnia with regards to different age groups, gender, duration of CKD, and the number of hemodialysis episodes. The Fisher-exact test suggested that insomnia was associated with hemodialysis (p-value 0.000). However, it did not show any significant correlation with the age of the subjects, and the p-value was found to be 0.362 (Table 2).

**Table 2:** Correlation of age group and hemodialysis sessions with insomnia

Age Group Category	Patients with Insomnia	p-value
18 to 30 years	9(34.6%)	0.396
> 30 to 40 years	19(65.4%)	
>40 to 50 years	14(45.2%)	
> 50 to 60 years	12 (27.3%)	
HEMODIALYSIS		
11-15	81(54.7%)	0.000
>15-20	12(8.1%)	
>20-25	55(37.2%)	

## Discussion

The present study suggests that insomnia can be frequently observed among patients with CKD. Nearly one-third of our patient group were diagnosed with insomnia, whereas several studies have shown a wide range of prevalence ranging from 54% to 69%. Therefore, the need to adopt more effective treatment strategies for CKD insomniacs is of paramount importance [13].

Murtagh et al. published a systemic review of 17 studies, where he reported 44% of the patients had sleep disturbances with underlying co-morbid ESRD [14]. The purpose of the current study is to highlight the significance of insomnia and other sleep-related conditions because they are under-recognized by renal healthcare providers as reported by Weisbord et al. in his descriptive study [15].

Sabbatini et al. found a higher prevalence of insomnia among females and elderly patients [16]; this is in contrast to our study, as we report a higher number of patients with insomnia in the age group of 30-50 years. The most common reason for insomnia in our study was difficulty in staying asleep and early morning awakening, whereas Shahbaj et al. reported problems in sleep initiation and sleep maintenance as more prevalent causes for Insomnia [17].

CKD is associated with diabetes, depression, and increased cardiovascular mortality. Consequently, insomnia ensues secondary to diabetes and depression [18]. Insomnia in CKD subjects could be due to various factors: money spent on therapy, co-morbid sleep disorders like restless leg syndrome (RLS) and obstructive sleep apnoea, and risk factors such as diabetes and anxiety [19]. However, we did not investigate the financial burden among subjects; it could be one of the primary reasons for insomnia among third-world countries.

Diabetes and cardiovascular diseases are significant causes of insomnia, as reported by Manan et al. [17], but we could not assess causality due to the cross-sectional nature of the study. A descriptive study published by Mark et al. assessed sleep quality among patients with insomnia and reported three times more risk to sleep less than five hours per night and more than half had difficulty getting back to sleep or waking up too early in the morning or feeling tired and not getting ample amount of sleep [20]. In addition, he reported insomnia in 50%-80% of patients on hemodialysis; the current study observed a similar trend between insomnia and hemodialysis. Rai et al. validated these findings by proposing that insomnia was present in 60.9% of patients on hemodialysis and significantly higher in patients on dialysis for more than one year (p=0.003) [21].

Sleep-related problems are often overlooked by physicians and often omitted by the patient due to various reasons such as negative approach by the family or patient towards psychiatric illnesses, lack of interdisciplinary communication and cooperation among medical specialties [22]. Melatonin is recommended for the regulation and improvement of the sleep-wake cycle in patients with insomnia, as it improves the subjective and objective parameters of sleep, with no consequent side effects [23]. Yang et al. reviewed around 12 randomized controlled trials (RCT) and one cohort study to analyze the effect of non-pharmacological treatment modalities (cognitive-behavioral therapy (CBD), acupressure, exercise) with insomnia, and concluded that CBD for insomnia is a recommendable treatment option for patients on hemodialysis [24].

Limitations

The limitations of our study include it being a single-center study with limited sample size. Information regarding the quality of life and financial aspects were not included in the study. Behavioral factors as causes of insomnia and their consequences on the quality of life were not reviewed. Furthermore, we did not evaluate various co-morbidities among CKD patients and did not investigate other sleep-related problems such as obstructive sleep apnea (OSA) and parasomnia.

Clinical recommendations

There is a need for practicing physicians to address sleep complaints in their patients with chronic kidney disease. We recommend an appropriate clinical evaluation that includes referral to a psychiatrist to evaluate insomnia and assess sleep hygiene in CKD patients.

Very often, the fundamental component of sleep hygiene is neglected, and relatively minor changes in patient behavior can lead to significant improvement in sleep quality. When this approach doesn't work clinicians should refer patients with CKD along with insomnia for cognitive behavioral therapy or medical therapy. Even though insomnia is prevalent among renal patients, nephrologists should be motivated to use the expertise of colleagues trained in sleep medicine and employ a multidisciplinary approach.

## Conclusions

In patients with ESRD, the diagnosis and management of insomnia is overlooked due to an overlapping presentation with CKD. The approach to reviewing this association is to consider sleep disorders as a secondary consequence of the primary disease. Another method is to segregate insomnia from concurrent medical/psychiatric problems and manage it as an independent co-occurring disorder. The high proportion of insomnia in the CKD population highlights the need to conduct extensive and well-organized clinical trials. Furthermore, multidisciplinary and multifactorial research would explain the complex interrelationship between sleep and kidney disease. Consequently, this will lead to the development of novel treatments for sleep abnormalities, which can reduce the psychological and physiological burdens of CKD patients on HD.
